# Supplementary material for: DNA barcoding of Austrian snow scorpionflies (Mecoptera, Boreidae) reveals potential cryptic diversity in Boreus westwoodi
Source: PeerJ. 2021 May 14;9:e11424. doi: 10.7717/peerj.11424 (PMC8127955; doi:10.7717/peerj.11424)
Supplement: Supplemental Information 3 [file peerj-09-11424-s003.pdf]

BOLD TaxonID Tree

Title	:	Tree Result - DS-BOREUS
Date	:	18-Mar-2021
Data Type	:	Nucleotide
Distance Model	:	Kimura 2 Parameter
Marker	:	COI-5P
Colourization	:	[blue]=Stop Codons [red]=Contamination or misidentification
Label	:	Sample ID
Label	:	Process ID
Label	:	Taxon
Label	:	Barcode Cluster (BIN)
Sequence Count	:	67
Species count	:	2
Genus count	:	1
Family count	:	1
Unidentified	:	0
BIN Count	:	7

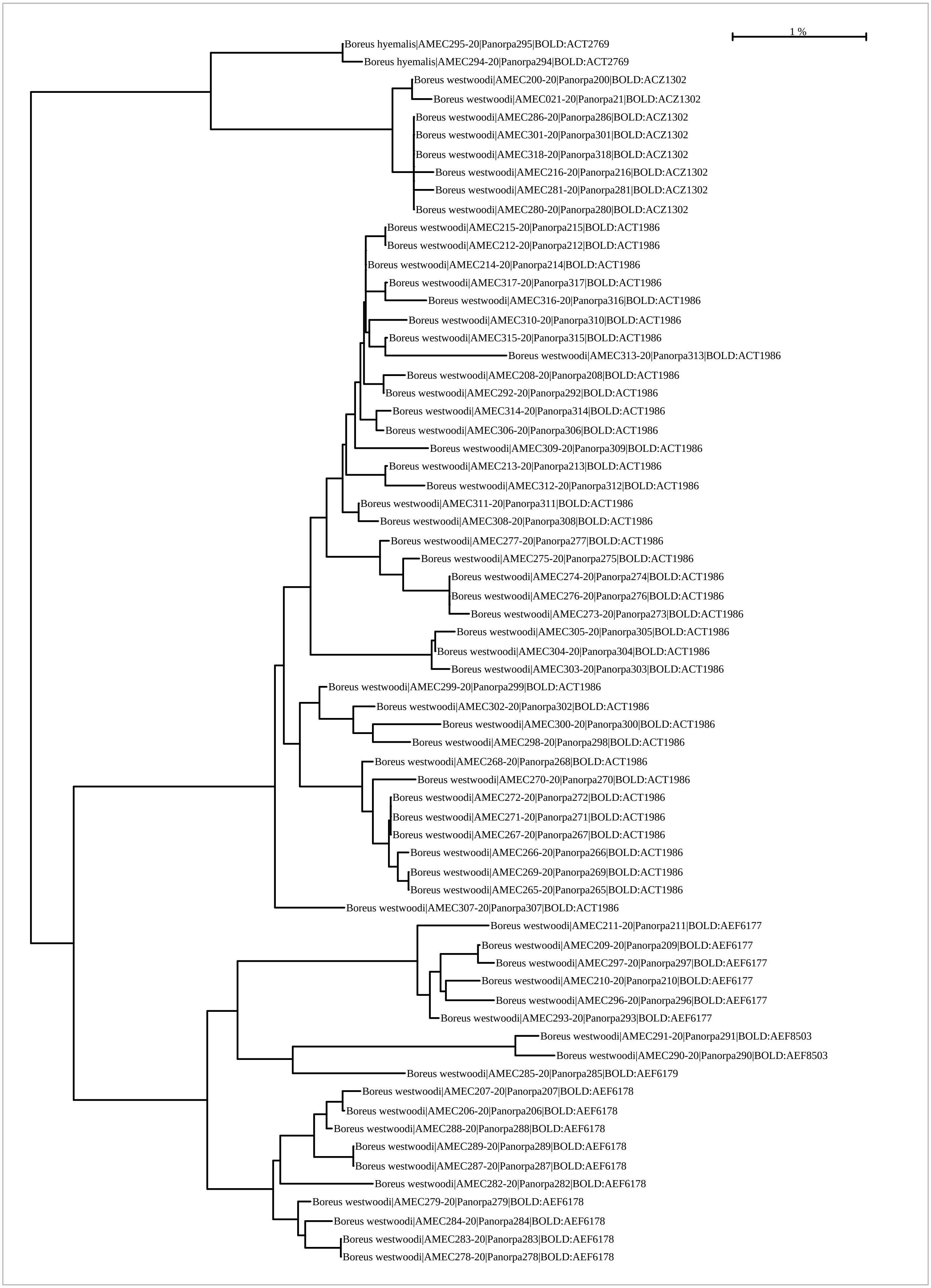
1 %
Boreus hyemalis|AMEC295-20|Panorpa295|BOLD:ACT2769
Boreus hyemalis|AMEC294-20|Panorpa294|BOLD:ACT2769
Boreus westwoodi|AMEC200-20|Panorpa200|BOLD:ACZ1302
Boreus westwoodi|AMEC021-20|Panorpa21|BOLD:ACZ1302
Boreus westwoodi|AMEC286-20|Panorpa286|BOLD:ACZ1302
Boreus westwoodi|AMEC301-20|Panorpa301|BOLD:ACZ1302
Boreus westwoodi|AMEC318-20|Panorpa318|BOLD:ACZ1302
Boreus westwoodi|AMEC216-20|Panorpa216|BOLD:ACZ1302
Boreus westwoodi|AMEC281-20|Panorpa281|BOLD:ACZ1302
Boreus westwoodi|AMEC280-20|Panorpa280|BOLD:ACZ1302
Boreus westwoodi|AMEC215-20|Panorpa215|BOLD:ACT1986
Boreus westwoodi|AMEC212-20|Panorpa212|BOLD:ACT1986
Boreus westwoodi|AMEC214-20|Panorpa214|BOLD:ACT1986
Boreus westwoodi|AMEC317-20|Panorpa317|BOLD:ACT1986
Boreus westwoodi|AMEC316-20|Panorpa316|BOLD:ACT1986
Boreus westwoodi|AMEC310-20|Panorpa310|BOLD:ACT1986
Boreus westwoodi|AMEC315-20|Panorpa315|BOLD:ACT1986
Boreus westwoodi|AMEC313-20|Panorpa313|BOLD:ACT1986
Boreus westwoodi|AMEC208-20|Panorpa208|BOLD:ACT1986
Boreus westwoodi|AMEC292-20|Panorpa292|BOLD:ACT1986
Boreus westwoodi|AMEC314-20|Panorpa314|BOLD:ACT1986
Boreus westwoodi|AMEC306-20|Panorpa306|BOLD:ACT1986
Boreus westwoodi|AMEC309-20|Panorpa309|BOLD:ACT1986
Boreus westwoodi|AMEC213-20|Panorpa213|BOLD:ACT1986
Boreus westwoodi|AMEC312-20|Panorpa312|BOLD:ACT1986
Boreus westwoodi|AMEC311-20|Panorpa311|BOLD:ACT1986
Boreus westwoodi|AMEC308-20|Panorpa308|BOLD:ACT1986
Boreus westwoodi|AMEC277-20|Panorpa277|BOLD:ACT1986
Boreus westwoodi|AMEC275-20|Panorpa275|BOLD:ACT1986
Boreus westwoodi|AMEC274-20|Panorpa274|BOLD:ACT1986
Boreus westwoodi|AMEC276-20|Panorpa276|BOLD:ACT1986
Boreus westwoodi|AMEC273-20|Panorpa273|BOLD:ACT1986
Boreus westwoodi|AMEC305-20|Panorpa305|BOLD:ACT1986
Boreus westwoodi|AMEC304-20|Panorpa304|BOLD:ACT1986
Boreus westwoodi|AMEC303-20|Panorpa303|BOLD:ACT1986
Boreus westwoodi|AMEC299-20|Panorpa299|BOLD:ACT1986
Boreus westwoodi|AMEC302-20|Panorpa302|BOLD:ACT1986
Boreus westwoodi|AMEC300-20|Panorpa300|BOLD:ACT1986
Boreus westwoodi|AMEC298-20|Panorpa298|BOLD:ACT1986
Boreus westwoodi|AMEC268-20|Panorpa268|BOLD:ACT1986
Boreus westwoodi|AMEC270-20|Panorpa270|BOLD:ACT1986
Boreus westwoodi|AMEC272-20|Panorpa272|BOLD:ACT1986
Boreus westwoodi|AMEC271-20|Panorpa271|BOLD:ACT1986
Boreus westwoodi|AMEC267-20|Panorpa267|BOLD:ACT1986
Boreus westwoodi|AMEC266-20|Panorpa266|BOLD:ACT1986
Boreus westwoodi|AMEC269-20|Panorpa269|BOLD:ACT1986
Boreus westwoodi|AMEC265-20|Panorpa265|BOLD:ACT1986
Boreus westwoodi|AMEC307-20|Panorpa307|BOLD:ACT1986
Boreus westwoodi|AMEC211-20|Panorpa211|BOLD:AEF6177
Boreus westwoodi|AMEC209-20|Panorpa209|BOLD:AEF6177
Boreus westwoodi|AMEC297-20|Panorpa297|BOLD:AEF6177
Boreus westwoodi|AMEC210-20|Panorpa210|BOLD:AEF6177
Boreus westwoodi|AMEC296-20|Panorpa296|BOLD:AEF6177
Boreus westwoodi|AMEC293-20|Panorpa293|BOLD:AEF6177
Boreus westwoodi|AMEC291-20|Panorpa291|BOLD:AEF8503
Boreus westwoodi|AMEC290-20|Panorpa290|BOLD:AEF8503
Boreus westwoodi|AMEC285-20|Panorpa285|BOLD:AEF6179
Boreus westwoodi|AMEC207-20|Panorpa207|BOLD:AEF6178
Boreus westwoodi|AMEC206-20|Panorpa206|BOLD:AEF6178
Boreus westwoodi|AMEC288-20|Panorpa288|BOLD:AEF6178
Boreus westwoodi|AMEC289-20|Panorpa289|BOLD:AEF6178
Boreus westwoodi|AMEC287-20|Panorpa287|BOLD:AEF6178
Boreus westwoodi|AMEC282-20|Panorpa282|BOLD:AEF6178
Boreus westwoodi|AMEC279-20|Panorpa279|BOLD:AEF6178
Boreus westwoodi|AMEC284-20|Panorpa284|BOLD:AEF6178
Boreus westwoodi|AMEC283-20|Panorpa283|BOLD:AEF6178
Boreus westwoodi|AMEC278-20|Panorpa278|BOLD:AEF6178
