# Supplementary figures and images for: DNA barcoding of Austrian snow scorpionflies (Mecoptera, Boreidae) reveals potential cryptic diversity in Boreus westwoodi
Source: PeerJ. 2021 May 14;9:e11424. doi: 10.7717/peerj.11424 (PMC8127955; doi:10.7717/peerj.11424)

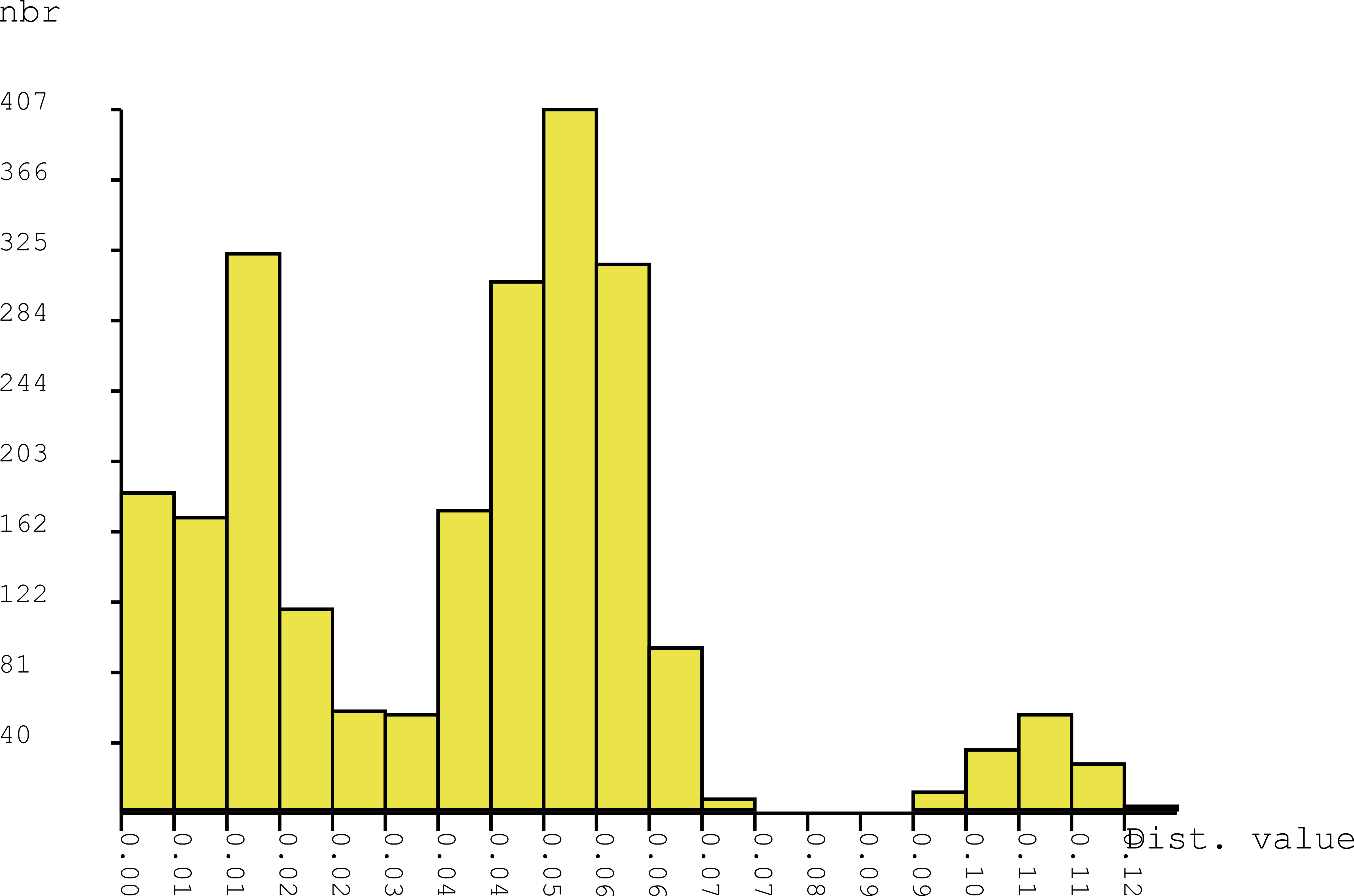

Supplement: Supplemental Information 4 [file peerj-09-11424-s004.png]
